# Plasticity of Mitochondrial DNA Inheritance and Its Impact on Nuclear Gene Transcription in Yeast Hybrids

**DOI:** 10.3390/microorganisms8040494

**Published:** 2020-03-31

**Authors:** Sarah K. Hewitt, Kobchai Duangrattanalert, Tim Burgis, Leo A.H. Zeef, Samina Naseeb, Daniela Delneri

**Affiliations:** 1 Manchester Institute of Biotechnology, Faculty of Biology, Medicine and Health, University of Manchester, Manchester M1 7DN, UK; sarah.hewitt@gmx.com (S.K.H.); kduangrattanalert@gmail.com (K.D.); samina.naseeb@manchester.ac.uk (S.N.); 2 Division of Evolution and Genomic Science, Faculty of Biology, Medicine and Health, University of Manchester, Manchester M13 9PL, UK; tim.burgis@gmail.com (T.B.); Leo.Zeef@manchester.ac.uk (L.A.H.Z.)

**Keywords:** nuclear transcription, hybrids yeast, mitochondria

## Abstract

Mitochondrial DNA (mtDNA) in yeast is biparentally inherited, but colonies rapidly lose one type of parental mtDNA, thus becoming homoplasmic. Therefore, hybrids between the yeast species possess two homologous nuclear genomes, but only one type of mitochondrial DNA. We hypothesise that the choice of mtDNA retention is influenced by its contribution to hybrid fitness in different environments, and the allelic expression of the two nuclear sub-genomes is affected by the presence of different mtDNAs in hybrids. *Saccharomyces cerevisiae/S. uvarum* hybrids preferentially retained *S. uvarum* mtDNA when formed on rich media at colder temperatures, while *S. cerevisiae* mtDNA was primarily retained on non-fermentable carbon source, at any temperature. Transcriptome data for hybrids harbouring different mtDNA showed a strong environmentally dependent allele preference, which was more important in respiratory conditions. Co-expression analysis for specific biological functions revealed a clear pattern of concerted allelic transcription within the same allele type, which supports the notion that the hybrid cell works preferentially with one set of parental alleles (or the other) for different cellular functions. Given that the type of mtDNA retained in hybrids affects both nuclear expression and fitness, it might play a role in driving hybrid genome evolution in terms of gene retention and loss.

## 1. Introduction

Mitochondrial DNA (mtDNA) is inherited in a non-mendelian manner outside the nucleus. Hybrids between *Saccharomycetes* tend to carry mtDNA from just one parent i.e., they are homoplasmic. All the strains of *S. pastorianus*, a natural lager brewing yeast hybrid between *S. cerevisiae* and *S. eubayanus* [1], contain only mtDNA from the cryotolerant parent, *S. eubayanus* [2,3,4]. Other industrial *Saccharomyces* brewery hybrids also retain the non-*S. cerevisiae* mitochondrial genome [5,6]. The influence of environmental conditions on the inheritance of mtDNA in yeast and the impact of mtDNA on the fitness and transcription of yeast hybrids living and reproducing in different environments has been largely overlooked. The adaptation to temperature is one of the primary drivers of evolution in yeast [7] and it is responsible for much of the phenotypic divergence between yeasts, even allowing different strains to exist in the same ecological niche [8]. Thus, the environmental temperature that is present at the time of hybrid formation might play a role in mitochondrial genome choice, and this choice subsequently might affect the fitness of the organism. *Saccharomyces* yeast can both respire, which requires functioning mitochondria, or ferment, which does not. In fact, yeast can survive adequately without functioning mitochondria, and form smaller “petite” colonies. Thus, the type of carbon source present in the environment; i.e., a fermentable carbon source or a carbon source that can only be respired, might also affect mtDNA choice and the future fitness of the organism. 

We crossed haploid *S. cerevisiae* BY4741 (Sc) and the cryotolerant species *S. uvarum* NCYC 2669 (Su) under a range of temperatures and under either fermentation or respiratory conditions to better understand how environmental conditions may affect the inheritance of mitochondrial DNA and its impact on fitness and evolution of the hybrid genome [9]. 

We analysed the genome expression in different hybrid mitotypes and showed that the type of mitochondrial DNA present might create a nuclear allelic expression bias in different biological pathways that depends also on environmental conditions, given the fact that most mitochondrial proteins are nuclear-encoded. Our expression analysis also reveals how different mtDNA may be affecting the hybrid’s transcriptome and how these expression differences may partially explain the differences in fitness observed between hybrids harbouring different mtDNA. Furthermore, when comparing *S. cerevisiae* and *S. uvarum* alleles within the same hybrid background, and comparing alleles in hybrids versus their parents allowed for us to distinguish cis-regulatory and trans-regulatory differences, respectively.

## 2. Materials and Methods

### 2.1. Yeast Strains and Media

*Saccharomyces cerevisiae* BY4741 and BY4743 were obtained from ThermoFisher Scientific, Leicester, UK. A KanMX cassette was previously engineered into the neutral *AAD3* locus of *S. cerevisiae* BY4741 (*MAT**a** his3∆0 leu2∆0 met15∆0 ura3∆0*) in order to confer selectivity [10]. *Saccharomyces uvarum* NCYC 2669 was obtained from the National Collection of Yeast Cultures, Norwich, UK. The triploid hybrid used as a control strain for DNA content analysis was previously constructed (this lab) between *S. cerevisiae* BY4741 and *S. uvarum* NCYC 2669. The yeast strains were maintained on YPDA (2% (*w*/*v*) Bacto Peptone, 1% (*w*/*v*) Bacto Yeast Extract, 2% (*w*/*v*) glucose, and 2% (*w*/*v*) agar). Hybrid growth was assessed on either YPDA or YP + 2% (*w*/*v*) glycerol.

### 2.2. Construction of Hybrids

*S. uvarum* NCYC 2669 was sporulated and dissected, as previously described [11]. Individual *S. uvarum* NCYC 2669 mixed mating type spores and *S. cerevisiae* BY4741 *MAT***a** haploids were placed into physical contact for mating while using a Singer Instruments MSM micromanipulator on a surface of either YPD agar or YP + glycerol agar. The plates were incubated at 10 °C, 16 °C, or 28 °C until colonies of 2–3 mm were visible. Colonies were then replica plated onto SDA + G418 selective plates (0.17% (*w*/*v*) YNB without additional amino acids, 0.5% (*w*/*v*) ammonium sulphate, 2% (*w*/*v*) glucose, 2% (*w*/*v*) agar, and 300 µg/mL G418 geneticin). These conditions exclusively select for hybrids, which can both tolerate geneticin and grow in the absence of additional amino acids. Species-specific primers were used to check for the presence of *S. cerevisiae* and *S. uvarum* subgenomes within each putative hybrid colony [10]. 

### 2.3. Whole Genome DNA Extraction

The colonies were inoculated in YPD and grown at 30 °C, 200 rpm for 20 h. Genomic DNA was extracted from yeast cells while using Wizard Genomic DNA Purification Kit (Promega, Southampton, UK), according to the supplied protocol and rehydrated in H_2_O overnight at 4 °C. The presence of genomic DNA was assessed by gel electrophoresis on a 1% (*w*/*v*) agarose gel in TAE (40 mM Tris base, 20 mM acetic acid, and 1 mM EDTA) buffer. The DNA was quantified using a Nanodrop 1000 spectrophotometer (ThermoFisher Scientific, Wilmington, DE, USA).

### 2.4. DNA Content Analysis by Flow Cytometry

The cells were prepared for flow cytometry by growing them overnight at 30 °C. The cells were fixed using 95% ethanol, treated with 2 mg/mL RNAse (Sigma-Aldrich, St. Louis, MO, USA) for two hours at 37 °C and 5 mg/mL protease solution for 30 min at 37 °C, stained with 1 µM Sytox Green (ThermoFisher Scientific, Wilmington, DE, USA), and sonicated using a Diagenode Biorupter (Diagenode Corporation, Liège, Belgium) for 20 s on low power to disrupt clumps. Cells were analysed with 488 nm excitation on a Beckman Coulter CyAn^TM^ ADP flow cytometry machine (Beckman Coulter, CA, USA). Green fluorescence was collected at 523 nm. The data were analysed using Summit v4.3 software (Beckman Coulter, CA, USA).

### 2.5. Analysis of Mitochondrial DNA by Sanger Sequencing

The primers for the amplification of mitochondrial *COX2* sequence were previously available [12]. All of the other primers for amplifying *ATP6*, *ATP8*, *ATP9*, *COX1*, and *COX3* were designed using Primer3 [13] to amplify the sequence from both parental species (Table S7). The PCR products for *COX2* and *COX3* were subsequently analysed by RFLP. *ATP6*, *ATP8*, *ATP9*, *COX1*, *COX2*, and *COX3* were partially sequenced using Phusion High-Fidelity DNA Polymerase. The mitochondrial gene reactions consisted of 100 ng genomic DNA, 0.5 µM of each primer, 1X Phusion HF Buffer, 200 µM dNTPs, 0.5 µL Phusion DNA Polymerase, and up to 50 µL of H_2_O. The *COX2* and *COX3* genes were amplified, as described previously [12]. *COX1*, *ATP6*, *ATP8*, and *ATP9* were amplified, as follows: 98 °C for 30 s; 35 × (98 °C for 10 s, 52 °C for 30 s, 72 °C for 15–30 s per kb); final extension 72 °C for 5 min. Mitochondrial gene PCR products were purified using the Qiagen PCR Purification Kit and then quantified using a Nanodrop 1000 spectrophotometer (ThermoFisher Scientific, Wilmington, DE, USA). To assess the parental origin of the amplified mitochondrial genes, 1000 ng of each was digested with either 1 U *Hin*f I and *Hin*d III, (Roche Diagnostics, Basel, Switzerland) for *COX2*, *COX3*, respectively, along with 2.5 µL SuRE/cut Buffer H (10×) and up to 25 µL H_2_O for one hour at 37 °C. The PCR products and restriction enzyme digest products were analysed along with parental controls by electrophoresis on a 1.5% (*w*/*v*) agarose TAE gel to determine their parental origin.

### 2.6. Growth Assays

The parental strains and hybrids were inoculated in 5 mL YPD overnight, washed twice in distilled sterile H_2_O and resuspended in 5 mL H_2_O to an OD_600_ of 0.4. 1/10 dilutions of each culture were made up to a dilution of 1/10,000. 4 µL of each culture and their three dilutions were spotted on to the agar. The plates were incubated at 4 °C, 10 °C, 16 °C, or 28 °C. The photographs of each plate were recorded every 12 h while using a Bio-rad Geldoc XR transilluminator with the Bio-Rad Quantity One software (Bio-Rad, Hercules, CA, USA). Note that diploid parental *S. cerevisiae* BY4743 was chosen rather than the parental haploid BY4741, in order to provide a ploidy comparative to the hybrids. Cell morphology and size were analysed by using an automatic cellometer (Nexcelom Biosciences, Lawrence, MA, USA).

### 2.7. RNA Extraction and Sequencing

The total RNA was extracted from three biological replicates of *S. cerevisiae* BY4743 and *S. uvarum* NCYC 2669, and from one biological replicate each of three independently constructed hybrids of BY4741 × NCYC 2669 with *S. cerevisiae* type mtDNA and three independently constructed hybrids of BY4741 × NCYC 2669 with *S. uvarum* mitochondria that had been constructed at 28 °C on YPD. Total RNA was extracted using Trizol reagent (Thermo Fisher Scientific, Leicester, UK), as described previously [14]. The integrity of the RNA was checked on 1.5% agarose gel. The quantity and quality of RNA was assessed using a Nanodrop 1000 spectrophotometer (Thermo Fisher Scientific, Wilmington, DE, USA).

mRNA was sequenced on an Illumina Hiseq 2500 (Illumina, San Diego, CA, USA) platform. Library preparation and RNA-Seq was performed by the Genomic Technologies Core Facility at The University of Manchester, UK. A total of twelve samples was sequenced: three *S. cerevisiae* and three *S. uvarum* biological replicates; three independently constructed hybrids with *S. cerevisiae* mitotype (HMtSc); and, three independently constructed hybrids with S. *uvarum* mitotype (HMtSu) for each temperature tested.

### 2.8. Reverse Transcription and Real-Time Quantitative PCR 

A Tetro cDNA synthesis kit (catalogue no. BIO-65043, Bioline, London, UK) was used to synthesise cDNA using random primers, while following the manufacturer’s instructions. Two genes from each condition (YPD-28 °C, YPD-16 °C, and YP + glycerol-16 °C) showing significant transcriptional changes in RNAseq dataset were picked for further validation of the expression by real-time PCR. Real-time PCR was performed in a Roche thermocycler on the cDNA of hybrids and parental strains using the iTaq Universal SYBR Green Supermix (catalogue no. 1725121, BioRad, Deeside, UK). Reverse transcription and real time PCR was done, as described previously [14,15]. Table S8 shows the primers used for real time.

### 2.9. Assembly, annotation, and DE analysis

*S. cerevisiae* (UCSC SacCer3) and *S. uvarum* genomes [16] were used to create an artificial genome that was composed of both species. The sequenced single-end reads were mapped to this artificial genome using STAR mapper, only reporting reads that could be uniquely mapped to one place in the combined genomes. The number of reads mapped per *S. cerevisiae* and *S. uvarum* genes was counted with htseq. The counts were normalised to adjust for the total number of reads. DESeq [17] was used normalise data and to calculate differential expression and mean values for each strain. All the data plotted in the Figures are based on the average of the triplicate sets.

Orthologous relationships between the 6010 annotated *S. bayanus var. uvarum* (now known as *S. uvarum*) genes [16] and *S. cerevisiae* genes (SGD, www.yeastgenome.org) were calculated using Inparanoid [18]. It was shown that 5224 genes had a simple one-to-one orthologous relationship with a *S. cerevisiae* gene. A further 105 genes with a one-to-many relationship were assigned to best matched *S. cerevisiae* gene. These 5329 pairs of orthologs are the basis for the analyses that are presented here. The published assembly of *S. bayanus var. uvarum* [16] appears to contain a section of the mitochondrial genome that was misassembled into nuclear contig Sbay_6. This erroneous section of Sbay_6 has been replaced with a stretch of Ns. A list of nuclear encoded proteins that physically interact with mitochondrial genes was derived from the *Saccharomyces* Genome Database (SGD, www.yeastgenome.org).

### 2.10. Functional Annotation and Co-Expression Analysis

Gene ontology analysis was performed while using the DAVID functional annotation tools [19,20] with a fold-change of ≥ 2 at *p* ≤ 0.05.

A co-expression network was constructed, and clusters of genes/alleles were identified that showed similar expression patterns in the four testing conditions and the two hybrids, based on Pearson correlation of the allele expression profiles. The average path length of this network is 9.86, and the node degree ranges between 1 and 26 with significant fit to a power law distribution (exponent = 2.43). This indicates that the network has a scale-free property, which is common to most biological systems, including metabolic pathways and transcriptional regulation maps [20,21]. This also implies that the network has a modular structure in which genes/alleles are co-regulated in response to changes in medium, temperature, and mitotype [22].

## 3. Results and Discussion

### 3.1. Different Mitochondrial DNA is Retained in Saccharomyces Hybrids Depending on the Environments Where They Have Been Formed

We investigated the ability of *S. cerevisiae* and the cryotolerant *S. uvarum* to successfully form viable hybrids (here after termed Sc/Su hybrids), under rich media containing either glucose (fermentable) or glycerol (non-fermentable) as carbon source at 28 °C, 16 °C, or 10 °C. We carried out a minimum of 22 crosses per condition (total of 202 crossings) and obtained 82 viable hybrids in total. *S. cerevisiae* and *S. uvarum* subgenomes in the hybrids was confirmed while using species-specific primers (Figure S1). The DNA content (2n) of the hybrids was confirmed by Sytox Green staining of DNA and analysis by flow cytometry (Figure S2). Tetrad dissection of the Sc/Su hybrids (384 spore in total) showed no viable spores, as expected for diploid hybrids (data not shown). All of the constructed hybrids had the same morphology and cell sizes ranging between 5.1–5.4 μm (Table S1).

The percentage of successful hybridisation from crosses on YPD at 28 °C was just below 100% (Table 1). With each decrease in temperature, there was a sharp decrease in the percentage of hybrids that were successfully formed. Overall, the percentage of viable crosses that were generated on YP + glycerol was lower than those that were generated on YPD. With each decrease in temperature there was a decrease in the number of crosses that resulted in viable hybrids, like crosses made on fermentative media (Table 1). The lower success of hybridization with decreasing temperature may be due to spores producing mating factors more slowly, thus reducing the overall likelihood of mating. 

Sanger sequencing of six mitochondrial genes *ATP6, ATP8*, *ATP9*, *COX1*, *COX2*, and *COX3* was carried out to determine which parental mtDNA was retained by each newly-constructed Sc/Su hybrid (File S1). Furthermore, *COX2* and *COX3* were also analysed by restriction length fragment polymorphism (RFLP analysis). When hybridisation occurred on YPD at 28 °C and 16 °C, there was no significant difference between the number of hybrids that retained *S. cerevisiae* mtDNA and the number that retained *S. uvarum* mtDNA. Strikingly, on YP + glycerol, at 28 °C and 16 °C, all hybrids retained their mtDNA from *S. cerevisiae*. Therefore, our results would indicate that *S. cerevisiae* mtDNA is preferentially inherited over *S. uvarum* mtDNA at warmer conditions, where respiratory ability is crucial. These findings conform to the hypothesis by Albertin and coworkers, who predicted using *in silico* modelling that a 1:1 mixed population of *S. cerevisiae* and *S. uvarum* mitotypes in respiratory conditions would, after several generations, be dominated by the *S. cerevisiae* mitotype [23]. At 16 °C, we also obtained one hybrid with recombinant mtDNA displaying a *COX2* restriction pattern of *S. cerevisiae* and a *COX3* restriction pattern of *S. uvarum*, and this was not investigated further.

When hybridisation took place on YPD at 10 °C, the majority of hybrids retained *S. uvarum* mtDNA rather than *S. cerevisiae* mtDNA. The single hybrid successfully formed at 10 °C contained *S. uvarum* mtDNA. A previous study of *Cryptococcus neoformans* showed that temperature influenced the inheritance of mtDNA from the MATα or MATa parents, whereby lowering the temperature allowed increasingly fewer hybrids to inherit mtDNA from the MATα parent [24]. 

In terms of phenotypic fitness, recent studies by Li et al. (2019) and Baker et al (2019) showed that the temperature tolerance in hybrids of *S. cerevisiae*/*S. uvarum* and *S. cerevisiae*/*S. eubayanus* corresponds to the parent donating the mitochondria [25,26]. We also assessed the growth rate of our HMtSc and HMtSu hybrids, in YPD and YPD-Glycerol under a range of temperatures and confirmed previous studies (Figure S3). At warmer temperatures, HMtSc was fitter than HMtSu, at colder temperatures HMtSu was fitter than HMtSc, and at 16 °C the two hybrid mitotypes showed similar growth on both a respiratory and fermentable carbon source (Figure S3). Taken together, these data indicate that temperature plays a wider role in the likelihood of both hybridisation occurring and on the selection of the type of mitochondria that were retained that can be evolutionary advantageous. The impact on fitness can also be strain dependent, as it is been previously shown that hybrids between different strains of the same species can have different characteristics [23,27]. Under stressful conditions, chromosome aneuploidy, such as full or partial uni-parental disomy, can arise in the genome and also affect the fitness [28,29]. However, in this study, the hybrids have not been evolved or adapted to any particular stress, which makes disomy unlikely. 

### 3.2. RNA Sequencing Strategy and Hierarchy of Factors Affecting Transcriptional Responses

Yeast mitochondria are comprised of proteins that were derived from both mtDNA (8 protein coding genes) and the nuclear genome ca. 1000 genes [30]. It is largely unclear how the mitochondrial DNA present in yeast hybrids influences the transcriptional response of the nuclear genome. For transcriptome analysis, we chose six independently constructed (at 28 °C in YPD media) hybrids of *S. cerevisiae* and *S. uvarum*, three with *S. cerevisiae* mtDNA and three with *S. uvarum* mtDNA. These hybrids, along with the parental strains, were grown in rich media either containing glucose (YPD) or glycerol (YP-glycerol) as carbon source at 28 °C, a temperature at which there was a significant phenotypic difference between mitotypes, and at 16 °C, where both mitotypes showed a more similar fitness profile.

A strategy was designed to perform gene expression analysis of each subgenome in the hybrid strains. Firstly, the reads were mapped to a combined genome index of *S. cerevisiae* (SacCer3) and *S. uvarum* (adapted from [16], only counting reads that mapped perfectly once to the combined genomes (Figure S4). The reads that were not uniquely mapped were discarded because it could not be established which subgenome they had originated from. The same workflow was applied to the parental strains to ensure that differential expression between all samples was not biased by excluding ambiguous reads. Secondly, reads were only counted for 5229 genes that had a clear one-to-one orthologous relationship between the two parental strains.

**Figure 1:**
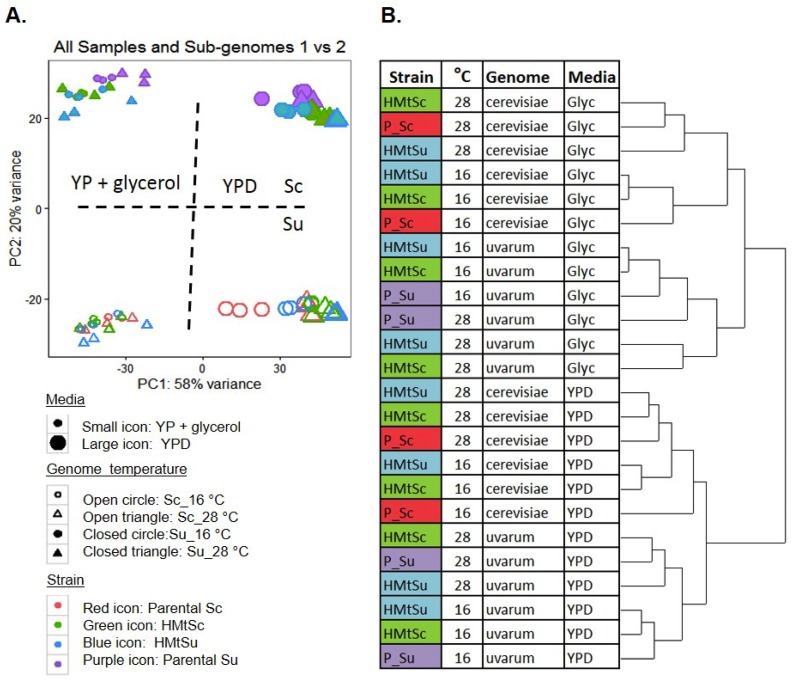
Clustering of expression data from RNAseq of hybrids and parental strains in different growth conditions. (**A**). PCA analysis of the transcriptome of each subgenome of HMtSc and HMtSu, and the parental strain BY4743 *S. cerevisiae* and *S. uvarum* strain NCYC 2669. The expression data separates primarily by growth media (YP + glycerol or YPD), and secondly by subgenome. Dotted lines show the approximate main separation of clusters along the first and second principal components. (**B**). Dendrogram describing more detailed clustering of strain and temperature within each growth condition.

Bioinformatic integration of gene expression from these two subgenomes was successful, based on a PCA analysis of the whole dataset. These data show that the biological factor of media type was the strongest contributor to gene expression, separating on the first component, whilst *S. cerevisiae* and *S. uvarum* subgenomes separate on the second principal component (Figure 1A). Gene expression data were clustered to visualize the hierarchy of effects of the four experimental factors (medium, genome, temperature, strain) (Figure 1B). Generally, within each subgenome, temperature has a greater impact on gene expression than the genetic background (hybrid- or parent-derived subgenome). 

The subgenomes of the hybrids with different mtDNA cluster closer together than they do with either parent, with two exceptions: *i.* the *S. cerevisiae* subgenome of HMtSc in YP + glycerol at 28 °C clusters with its Sc parent; and, *ii.* the *S. uvarum* subgenome of HMtSc in YPD at 28 °C clusters with its Su parent. This indicates that, in general, medium, subgenome type, and temperature have a greater influence on gene expression than the type of mtDNA in a hybrid, and the expression of a given subgenome will be more similar between the mitotypes than between hybrids and parent.

The verification of RNAseq data was carried out via real time PCR by validating the expression of two genes from each condition (i.e., YPD-28 °C, YP + glycerol-28 °C, and YPD-16 °C; Figure S5). The transcription of Sc and Su alleles was checked in both hybrids HMtSc and HMtSu. The expression levels of tested genes from the RNAseq and the real time PCR showed that there was concordance between both of the methods.

### 3.3. Plasticity of Allele Specific Expression in Sc/Su Hybrids

**Figure 2:**
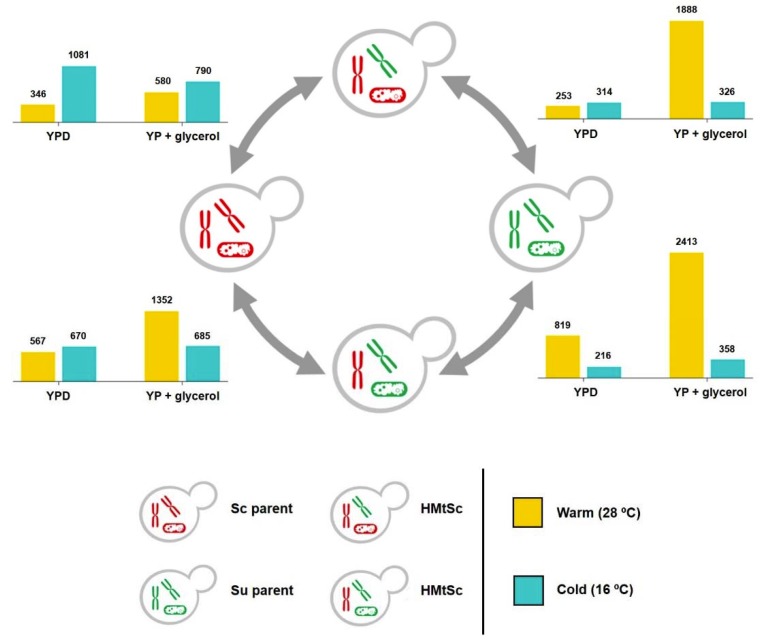
Differential expression between parental strains and hybrids. The number of differentially expressed genes between parental strains and the corresponding hybrid subgenome in HMtSc and HMtSu, grown in different conditions (*p* value < 0.05, logfc > ±1). Yellow bars denote growth conditions of 28 °C and blue bars denote growth conditions of 16 °C. YPD, fermentable carbon source; YP + glycerol, respiratory carbon source. Sc, *S. cerevisiae*; Su, *S. uvarum*.

We investigated how strongly the hybrid background, in relation to the environment, affects the expression levels of the Sc subgenome and Su subgenome. Specifically, we compared the gene expression of *S. cerevisiae* or *S. uvarum* alleles in the hybrids with their original expression profile in the respective parental backgrounds, within four growth conditions (Figure 2 and Table S2). The proportion of genes that were differentially expressed between parental strains and each hybrid greatly depended on growth condition and the mtDNA background (mitotype) of the hybrid. For example, there was a greater level of differential expression between the parental *S. cerevisiae* and the Sc alleles in HMtSc in cold conditions than warm conditions, regardless of carbon source (Figure 2 and Table S2). These expressional changes at cold are, however, mitigated in the HMtSu background. On the other hand, a higher proportion of genes were differentially expressed between parental *S. uvarum* and the Su alleles in HMtSc in the warm respiratory condition when compared to the cold condition, and these changes were exacerbated in the HMtSu background (Figure 2 and Table S2). 

Overall, these data show that, when comparing hybrids with parents, the *S. uvarum* subgenome was more differentially expressed at warm temperatures, whilst the *S. cerevisiae* subgenome was more differentially expressed at cool temperatures. *S. cerevisiae* and *S. uvarum* alleles both showed a high degree of expression plasticity between the parental cells and hybrids, implying that the expression in hybrids might be subject to different trans-acting effects from each parental genome [31,32]. By this explanation, the effects of trans-acting regulation depended heavily on the environmental condition: *S. uvarum* alleles exerted a greater effect on the expression of *S. cerevisiae* allele in cool conditions than in warm conditions, whilst *S. cerevisiae* alleles exerted a greater effect on the expression of *S. uvarum* alleles in warm conditions than in cool conditions. Additionally, or alternatively, the gene expression differences between parental strains and hybrid subgenomes may be due to differences in gene dosage from either the *S. cerevisiae* or *S. uvarum* genome when comparing diploid parents to the equivalent haploid subgenome in hybrids.

**Figure 3:**
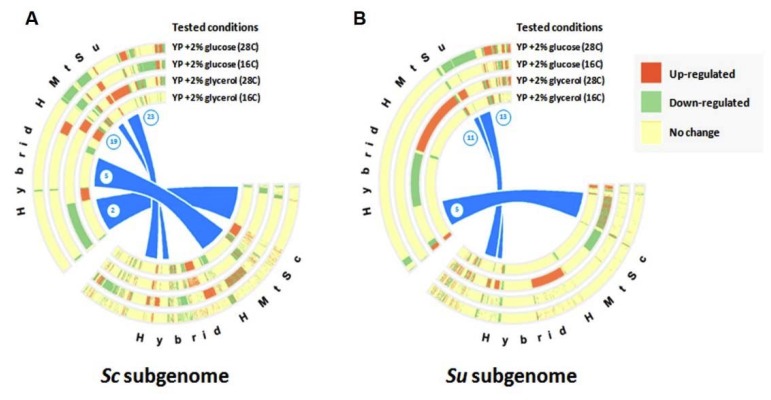
Circular heat maps representing differential gene expression and preservation of such changes in yeast hybrids versus parent strains. Circular heat map for the (**A**) *S. cerevisiae* (Sc) subgenome and (**B**) *S. uvarum* (Su) subgenome. Three different colours represent differential expression: red, up-regulation; green, down-regulation; light yellow, no significant change in gene expression. Heat maps of the two hybrids are connected to indicate divergence in differential expression of genes in two different mitotypes.

We determined whether any groups of differentially expressed alleles were enriched for genes that belonged to specific functional categories (Figure 3 and Table S3). Based on co-clustering statistics, we found that a proportion of gene expression changes between the hybrids and parents were common between the two mitotypes and, therefore, likely to be caused by the hybridisation event itself, for example, fitness changes would lead to impaired growth rates and concomitant transcriptional changes [33,34]. Transcriptional shock upon hybridisation has been documented in various plants and animals, and is interestingly associated with differentiation of phenotypic traits in a wide range of environments [35,36,37,38]. However, for those genes whose expression was significantly different between the two mitotypes (when compared to parents), we performed an enrichment analysis to determine the key molecular pathways that are affected by the mitochondrial DNA (Table S4). The differential gene expression and preservation in the hybrids HMtSc and HMtSu was represented as circularised heat maps (Figure 3). For the *Sc* subgenome (Figure 3A) and Su subgenome (Figure 3B), we found four and three clusters, respectively, containing alleles that were differentially expressed between the parents and hybrids that also significantly differed between mitotypes. Specifically, in the hybrid HMtSu in YP + glycerol at 28 °C, the Sc alleles within cluster 2 (Figure 3A) contained down-regulated genes involved in cell wall remodelling (i.e., actin cortical patch and downstream signalling) and the Sc alleles within cluster 23 (Figure 3A) contained up-regulated genes that are involved in cytoplasmic translation and mitochondrially-related functions (Table S4). It is known that the actin cytoskeleton plays an important role in several processes including endocytosis, cytokinesis, and cell morphology, and a role has been suggested in the regulation of cytoplasmic translation and mitochondrial function [39,40]. 

In cluster 2, we also observed up-regulation of *RAS2*, *TPK2*, *PKH1*, *MSN2*, *SSK2*, and *SLT2* genes, which are responsible for the propagation of stress signals along RAS and MAPK pathways [41,42]. This suggests that the hybrid HMtSu is experiencing starvation, oxidative shock, and cell wall and membrane stresses when grown in YP + glycerol at 28 °C. Despite cells trying to resolve the situation by promoting mitochondrial functions via actin and RAS signalling, Su mitochondrion appears to be less functional in the hybrid background when grown in warm conditions on a non-fermentable carbon source. 

In YP + glycerol at 28 °C, the Su alleles within cluster 5 (Figure 3B) only contain differentially regulated genes in the HMtSc hybrid, while, in the HMtSu background, no alteration of expression was detected. The majority of these genes are involved in ribosome biogenesis and mitotic cell cycle control (Table S4), which can be attributed to changes in growth rate [34,43].

We compared the patterns of gene expression directly between mitotypes HMtSc and HMtSu, by separately carrying out differential expression (DE) analysis on the *Sc* and *Su* subgenomes (Table S5). The identification of any differences aimed to clarify how harbouring different mtDNA influenced the expression of each subgenome within yeast hybrids. Strikingly, when directly comparing the expression profile of mitotypes, of the changes detected (*p* value < 0.05 logfc > ±1) a large proportion of the total alleles were far more differentially expressed at 28 °C, rather than 16 °C, particularly between the two *S. uvarum* subgenomes, and particularly in YP + glycerol (Table S5), indicating that the type of mtDNA that is harboured by the hybrid is extremely important for the nuclear transcription. These data parallels phenotypic data where mitotypes were most separate in terms of growth at 28 °C, when compared to 16 °C. In YPD at 28 °C, the type of mtDNA in the hybrid influenced the expression of the *S. uvarum* genome more than the expression of the *S. cerevisiae* subgenome. There were very few alleles that were significantly differentially expressed between mitotypes when hybrids were grown at colder temperature, particularly respiratory conditions. 

Overall, our study shows that allele preference is probably more important in respiratory conditions than fermentation conditions, given the larger number of genes that were differentially expressed YP-glycerol. In both mitotypes, there is also a generally a stronger allele preference at 28 °C than at 16 °C within any given media. Strikingly, in YP + glycerol at 16 °C, over one-third of genes with a preference for the *S. cerevisiae* allele in both hybrids are mitochondrial-associated. A study of allele preference in crosses of *Arabidopsis thaliana* and *A. lyrata* revealed a strong preference for the expression of *A. lyrata* alleles [44]. Almost 90% of genes that showed allele specific expression had preference for *A. lyrata*, which was likely caused by genome specific differences in epigenetic silencing. We found that different environmental conditions promoted differences in allele preference between hybrid mitotypes, but less strongly between sub-genomes. The identification of preferred parental mitochondrial-associated alleles is particularly relevant in our study, where mtDNA inheritance in hybrids greatly impacts fitness under different conditions.

### 3.4. Co-Expression Analysis in HMtSc and HMtSu Hybrids

**Figure 4:**
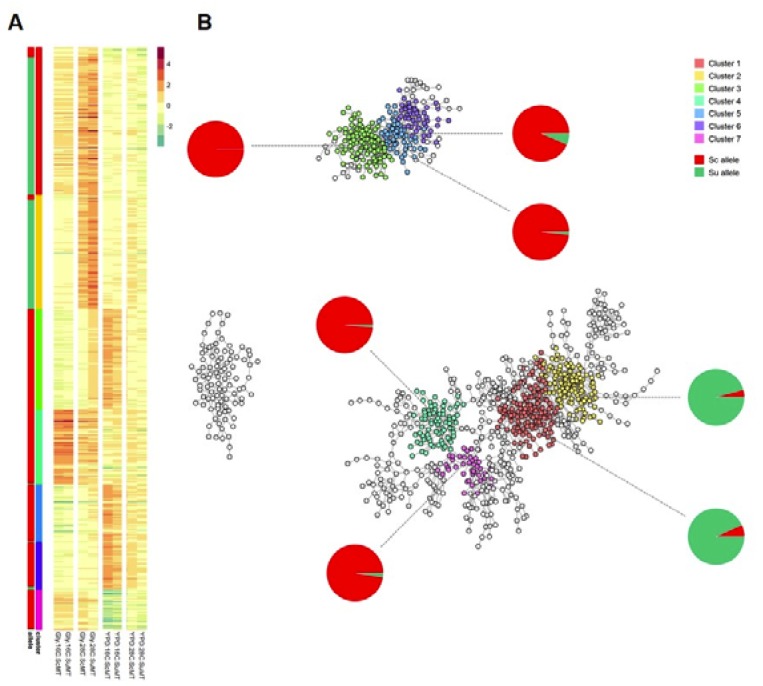
Analysis of allelic co-expression. (**A**) Heatmap was constructed based on significant correlation between alleles (i.e., Pearson coefficient ≥ 0.98 and *p*-value ≤ 0.05). Rows represent alleles of different genes and columns represent conditions used in this study. Each cell represents similarity in expression profiles with respect to the parental controls. Coloured bars show clusters identified by walktrap algorithm (step size = 13), which is implemented in the R package igraph, and proportion of Sc (red) and Su (green) alleles in the clusters. (**B**) A graphical representation of the co-expression map. Nodes represent alleles of different genes, and edges represent similarity in expression across the four testing conditions and the two hybrids. Each cluster is complemented with a pie chart indicating the proportion of Sc (*S. cerevisiae*, red) and Su (*S. uvarum*, green) alleles.

We further examined the relationship between alleles by building a co-expression network and performing an unsupervised learning to cluster alleles with similar expression profiles across the four tested conditions (YP + 2 % glucose and YP + 2 % glycerol at 28 °C and 16 °C) and the two mitotypes (HMtSc and HMtSu). The network has around 1000 nodes and 3200 edges with its topology being very close to scale-free (Figure 4). This topology is widely observed in biological systems, such as metabolic and regulatory networks. Functional enrichment analysis was performed in order to understand how allelic usage is associated with biological processes (Figure 4 and Table S6). The seven main clusters that showed concerted expression levels are enriched for mitochondrial and ribosomal genes modules (Figure 4B). 

Alleles clustered based on similar levels of transcription showed an uneven distribution of Sc or Su alleles across different transcription groups. Clusters with major functions in endoplasmic reticulum membrane-related process, lipid biosynthesis, and redox processes are significantly enriched with *S. uvarum* alleles (more than 90%; cluster 1 and cluster 2 Table S6), and clearly up-regulated in YP + glycerol medium at 28 °C with higher levels of expression in the HMtSu background. Genes that were involved in mitochondrial translation were upregulated in YP + glycerol medium at 16 °C. These findings indicate the importance of these biological processes for the metabolism of non-fermentable carbon source, particularly at higher temperatures. Additionally, it is possible that such an increase in differential expression of the Su alleles in these clusters might help to explain the fitness differences observed on solid media under the same conditions.

The higher number of Sc alleles in clusters 3, 5, and 6 has concerted differences. These clusters are strongly associated with ribosome biogenesis and non-coding RNA processing which, interestingly, are up-regulated in the HMtSc background at 16 °C. These data confirms studies by other research groups, where, for example, it has been demonstrated that ribosome biogenesis is intrinsically cold-sensitive, as mutants with deleted genes that are related to this function are unable to grow efficiently at low temperatures [45]. Therefore, carrying Sc mitochondria might confer disadvantages to cold adaptation, as certain genes were differentially regulated to deal with stress that is imposed by both mitochondria and temperature. These results are in agreement with the phenotypic data, as the way yeast hybrid fitness is affected by cold conditions is akin to the growth defect in mutants with ribosome biogenesis malfunction. Cluster 4, which encompasses mitochondrial inner membrane-associated genes, has a high number of Sc alleles that were strongly upregulated in YP + glycerol at 16 °C, and in YPD at 16 °C in both hybrids. Cluster 7 was enriched for mitochondrial translation function for Sc alleles and it was largely downregulated in YPD at 16 °C in both hybrids.

Surprisingly, of the known genes that were involved in mito-nuclear incompatibilities [46,47], only *MRS1* (YIR021W) shows differential expression between HMtSc and HMtSu.

Overall, here we show that for specific biological functions the transcriptional regulation was nearly exclusively retained within the same allele types, which could potentially be explained by the divergence of cis-regulatory elements and mitochondrial genome of the two related yeast species. Assume that this regulatory variation is essential for optimal growth within their specific environments, combining the two genomes might increase the repertoire of functional abilities towards a wider range of stresses.

## 4. Conclusions

This study sheds light on the plasticity of yeast hybrids in response to a range of environmental conditions and provides evidence that the choice of mtDNA retained by newly-formed hybrids could be evolutionarily advantageous. We showed that the mitochondrial retention is dependent on the environment where the hybrids are formed, and that the different parental mtDNAs affect the nuclear gene expression in a distinct manner. In the hybrids with different mitotypes, there is a generally stronger allele preference and we showed that, for specific biological functions, the transcriptional regulation was nearly exclusively retained within the same allele types, which was likely due to the divergence of cis-regulatory elements and mitochondrial genome of the two related yeast species.

The identification of preferred parental mitochondrial-associated alleles supports the notion that mtDNA inheritance in hybrids greatly impacts fitness under different conditions, and may have a role in driving the evolution of the hybrid nuclear genome in terms of gene retention and loss.

Given that several yeast hybrids are used in fermentation industries, our findings may aid the development of industrially important strains and help to elucidate pattern of genome evolution and hybrid vigor.

## Figures and Tables

**Table 1:** Hybrids created from individual crosses under different environmental conditions.

Temperature	Media	Total Number of Crosses Made	Total Number of Hybrids Made	No. of Hybrids with Sc MtDNA	No. of Hybrids with Su MtDNA
28 °C	YPD	22	21	10	11
	YP+Glycerol	22	19	19 **	0 **
16 °C	YPD	26	19	13	6
	YP+Glycerol	22	5	5 *	0
10 °C	YPD	48	17	5 **	12 **
	YP+Glycerol	62	1	0	1

** *p* ≤ 0.01, * *p* ≤ 0.05, Exact binomial test of goodness-of-fit for significant difference of type of mitochondria retained in the hybrids.

## Supplementary Materials

The following are available online at https://www.mdpi.com/2076-2607/8/4/494/s1. **Figure S1**: Experimental design of hybrid construction and gel electrophoresis of PCR products specific to *S. cerevisiae* or *S. uvarum*. Two sets of species-specific primers were used concurrently: A pair of *S. cerevisiae* specific primers (5′ AACAAAGACTCGTCCTGCTG 3′ and 5′ CACAGTTAACGCTTGCTTCC 3′) and amplify a 496 bp DNA fragment on *S. cerevisiae* chromosome XIII while a pair of *S. uvarum* primers (5′ CAAGTGAAACGTGCTAATTCC 3′ and 5′ GTCCTCGCAAATTATGGCTAC 3′) amplify an 817 bp fragment in the same chromosomal region. Lane M, DNA marker Hyperladder 1 kb (Bioline, UK). Lane 1, *S. cerevisiae*; Lane 2, *S. uvarum*; Lanes 3-5 examples of hybrids. **Figure S2**: Analysis of hybrid ploidy by flow cytometry. The ploidy of newly-constructed hybrids was assessed by comparison of DNA content to three standards. Upper: DNA content of ploidy controls analysed by flow cytometry. N, haploid *S. cerevisiae*; 2N, diploid *S. uvarum*; 3N triploid hybrid. Lower: An example of the DNA content profile of a typical newly-constructed *S. cerevisiae* x *S. uvarum* hybrid used in this study. A.U. Arbitrary Units used as a comparative measure of fluorescence. **Figure S3**. Growth assays of hybrid mitotypes on fermentative (YPD) or respiratory (YP + glycerol) carbon sources. The growth of three biological replicates of each hybrid mitotype (hybrids with *S. cerevisiae* mitochondria (HMtSc) and hybrids with *S. uvarum* mitochondria (HMtSu) and the parental strains *S. cerevisiae* BY4743 (Sc) and *S. uvarum* NCYC 2669 (Su) on YPD (A) or YP + glycerol (B), at 28 °C, 16 °C, 10 °C and imaged every 12 h. Each sample was diluted 1/10 up to a dilution of 1/10,000, moving from left to right, from an initial OD600 of 0.4 (far left). Images show time point of greatest growth differentiation between strains. **Figure S4.** Strategy of RNA-seq data analysis to allow gene expression analysis of each subgenome in the hybrid strains. Reads were mapped to a combined genome index of *S. cerevisiae* (red colour) and *S. uvarum* (green colour) only allowing reads that mapped perfectly once to the combined genomes (i.e., D, E, d, e). Reads that were not uniquely mapped (i.e., A) were discarded. **Figure S5**. Validation of RNAseq data. The bar chart represents the validation of RNAseq data with real time PCR performed on a subset of genes showing changes in expression in RNAseq experiments on hybrids HMtSc and HMtSu. ΔΔCt method was applied to calculate the fold change in expression and actin was used as a house keeping gene. Black and grey bars represent the fold change expression obtained from RNAseq and qPCR, respectively. **File S1.** Mitochondrial gene sequences of *S. cerevisiae*, *S. uvarum* and hybrids. **Table S1.** Mean cell size of parents and hybrid strains. **Table S2.** Differential expression between hybrids versus parental strains. **Table S3.** Differentially expressed alleles enriched for specific functional categories. **Table S4.** Genes differentially expressed in the two mitotypes in different conditions. **Table S5.** Number of genes showing differential expression between HMtSc and HMtSu. **Table S6.** Co-expression analysis and functional annotation. **Table S7.** Primer sequences for mitochondrial genes. **Table S8.** Primer sequences for Real time PCR.
