# Supplementary figures and images for: Plasticity of Mitochondrial DNA Inheritance and Its Impact on Nuclear Gene Transcription in Yeast Hybrids
Source: Microorganisms. 2020 Mar 31;8(4):494. doi: 10.3390/microorganisms8040494 (PMC7232527; doi:10.3390/microorganisms8040494)

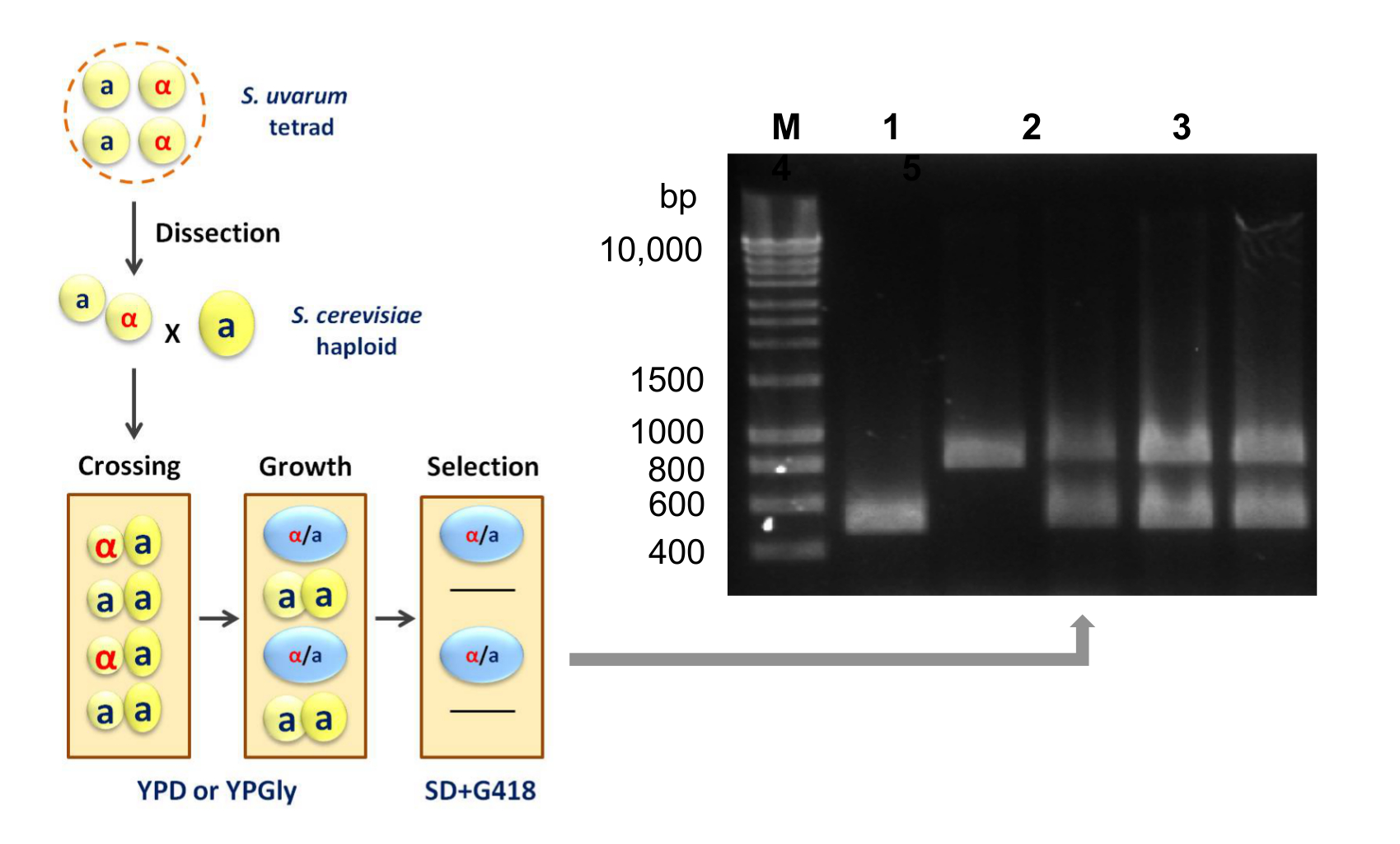

Supplement: Supplementary file 1 [file microorganisms-08-00494-s001.zip › microorganisms-754787-Supplementary material/Fig. S1.jpg]

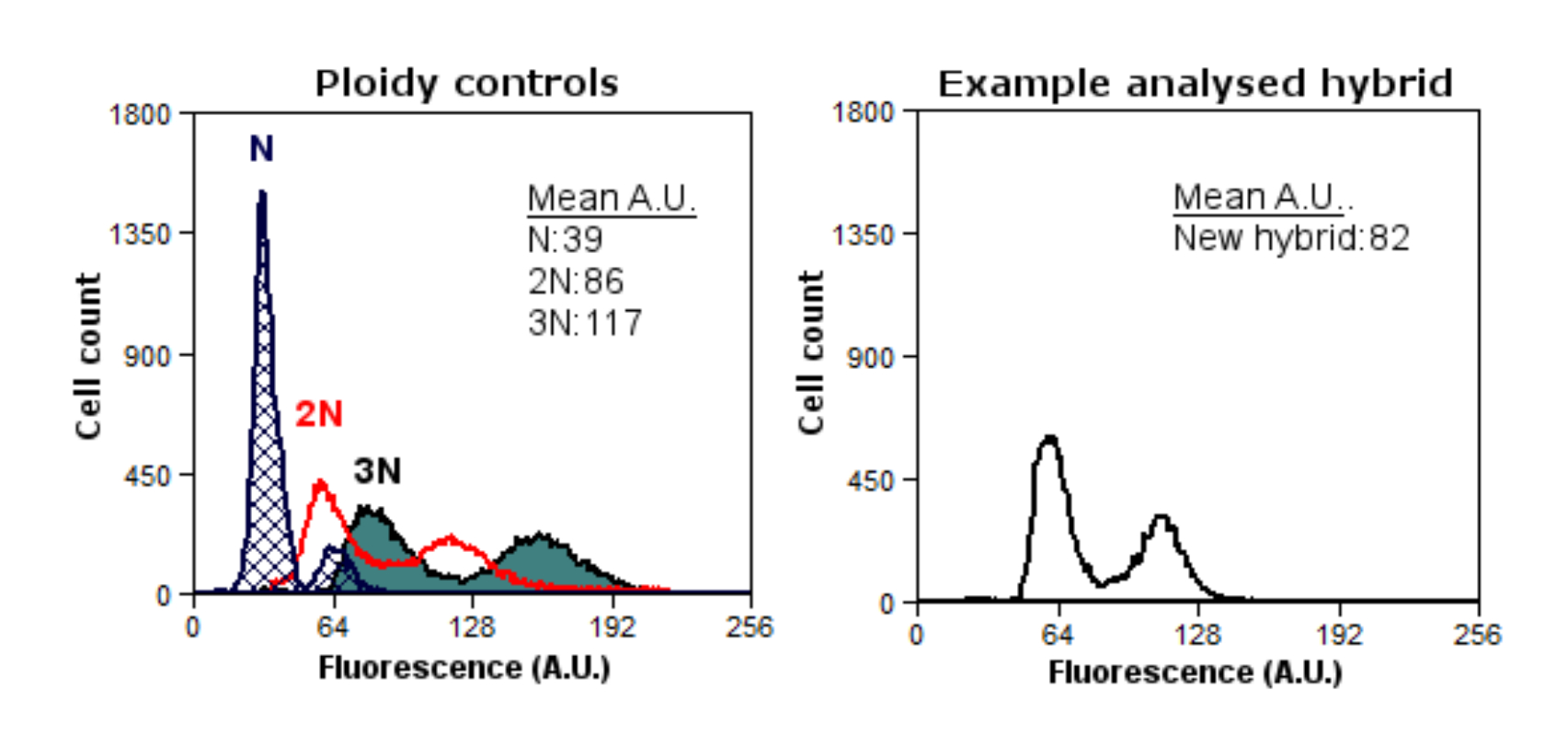

Supplement: Supplementary file 1 [file microorganisms-08-00494-s001.zip › microorganisms-754787-Supplementary material/Fig. S2.jpg]

## A. YPD

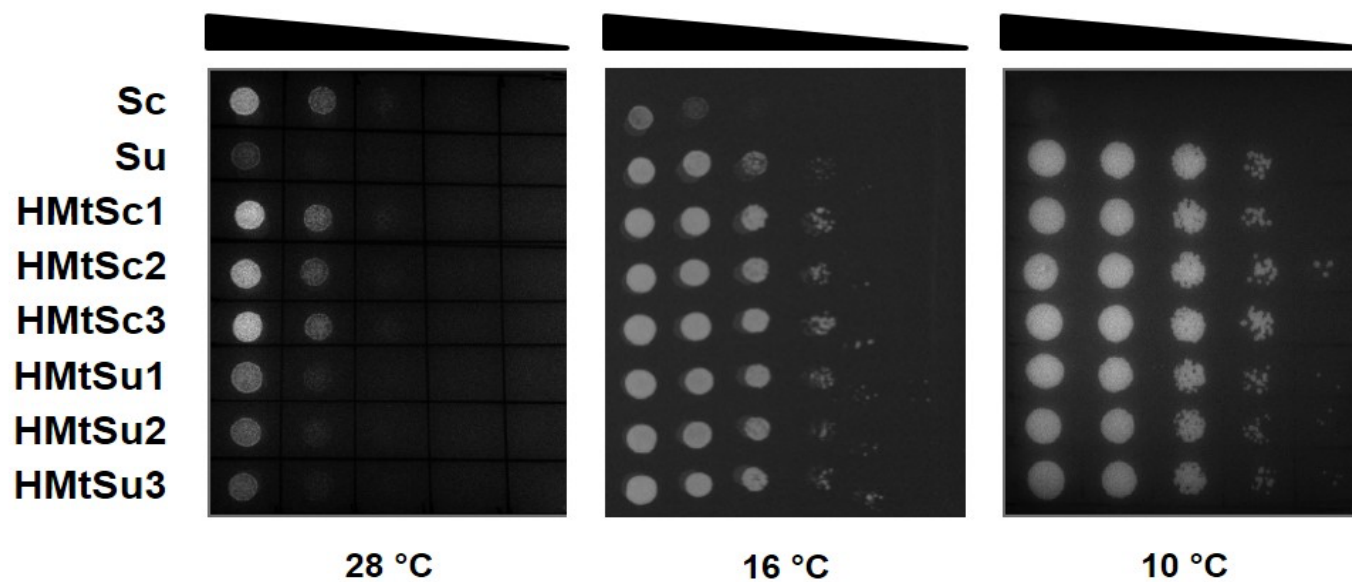

## B. YP + glycerol

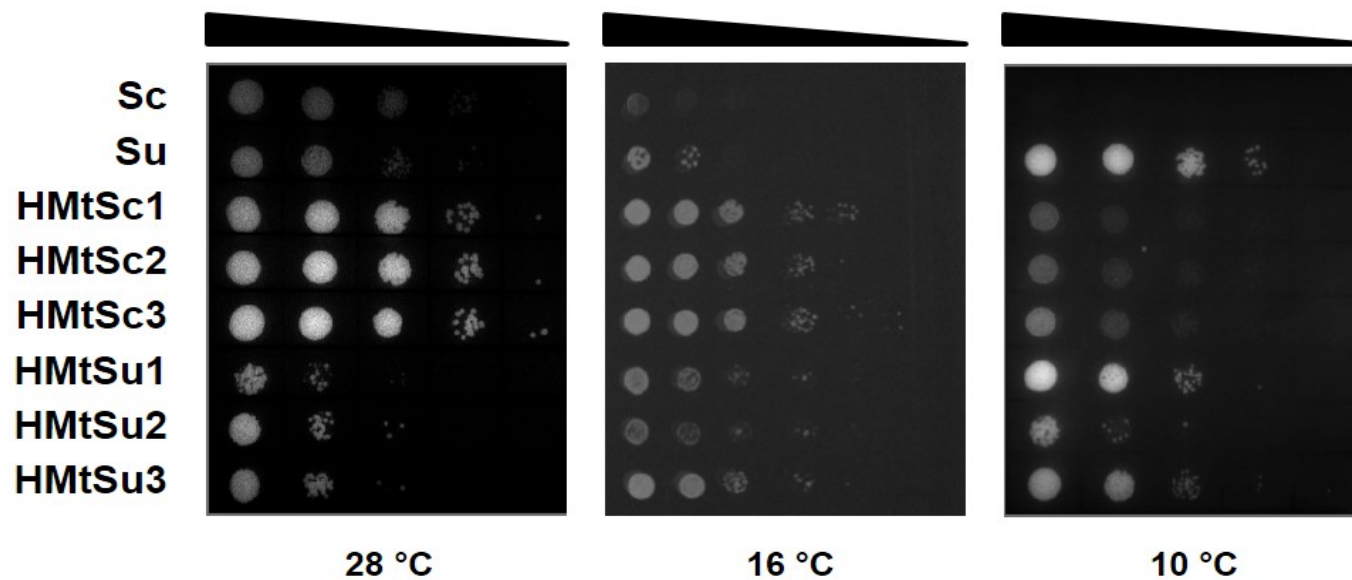

Supplement: Supplementary file 1 [file microorganisms-08-00494-s001.zip › microorganisms-754787-Supplementary material/Figure S3. Spot Assay in YPD 28 and 16oC.pdf]

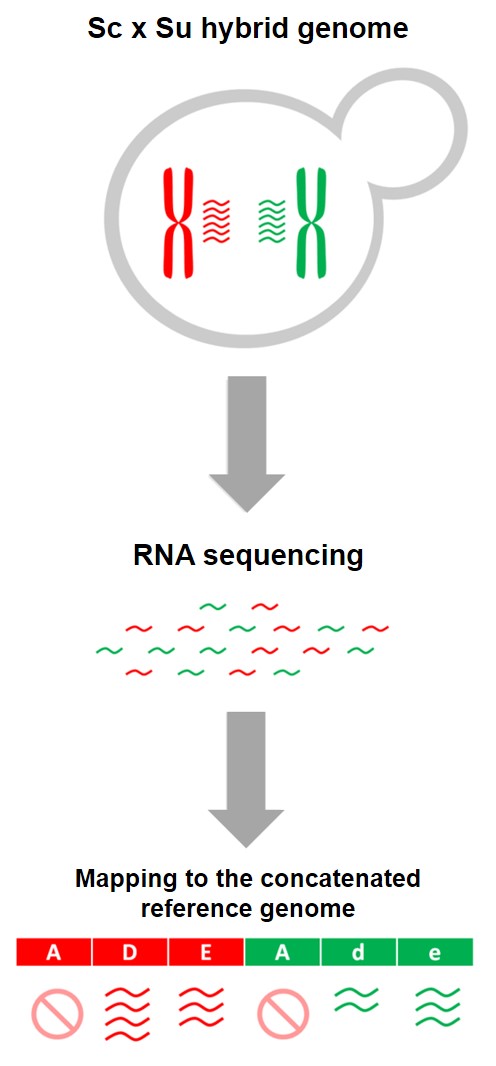

Supplement: Supplementary file 1 [file microorganisms-08-00494-s001.zip › microorganisms-754787-Supplementary material/Figure S4.jpg]

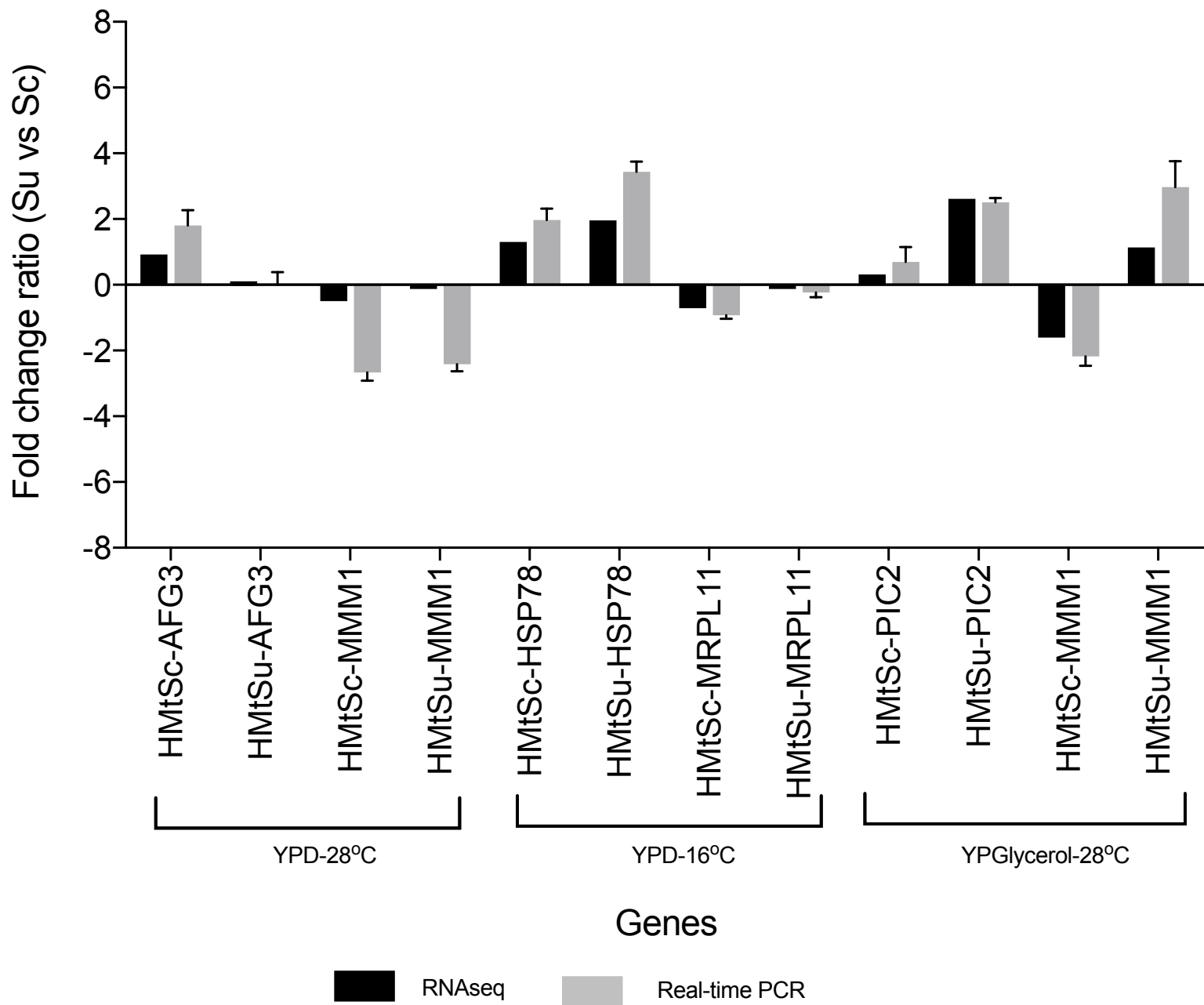

Supplement: Supplementary file 1 [file microorganisms-08-00494-s001.zip › microorganisms-754787-Supplementary material/Figure S5 with error bars.pdf]
